# Research Advances of the Autophagy‐Regulated Radiosensitivity

**DOI:** 10.1111/cpr.70056

**Published:** 2025-05-05

**Authors:** Hanyue Liu, Yanlan Xiao, Chuhao Dai, Keyu Chen, Xinyi Xu, Jianming Cai, Xuguang Hu, Jiaming Guo

**Affiliations:** ^1^ Department of Radiation Medicine, College of Naval Medicine Naval Medical University Shanghai China; ^2^ College of Basic Medicine Naval Medical University Shanghai China; ^3^ School of Public Health and Management Wenzhou Medical University Wenzhou China; ^4^ Department of Gastrointestinal Surgery Shanghai Changhai Hospital, Naval Medical University Shanghai China

**Keywords:** autophagy, metabolic regulation, oxidative stress, radiosensitivity, radiotherapy

## Abstract

Autophagy is an evolutionarily conserved process of cell self‐catabolism that provides a minimum level of energy for cellular homeostasis during metabolic stress. In radiotherapy (RT), it has been explicitly explained that autophagy plays a dual role in tumour control by tuning cellular radiosensitivity. However, the underlying molecular mechanism remains a conundrum. Therefore, it is of utmost importance to gain insight into the molecular mechanisms elaborating the autophagy‐mediated radiosensitivity and craft refined RT strategies for different tumours. Distinguishing it from previous reviews in the field, here we discuss the mechanisms of autophagy, especially its pro‐survival and growth‐suppressing mechanisms via regulation of radiosensitivity. We further outline some frontier RT adjuvant therapies targeting autophagy, in an endeavour to shed some light on the autophagy‐mediated pathways to harness radiosensitivity.

## Graphical abstract

Autophagy plays a dual role in regulating cellular radiosensitivity in radiotherapy (RT). We discuss the mechanisms by which autophagy promotes survival and growthsuppression by modulating radiosensitivity. It also outlines some frontier autophagy‐targeted radiotherapies.

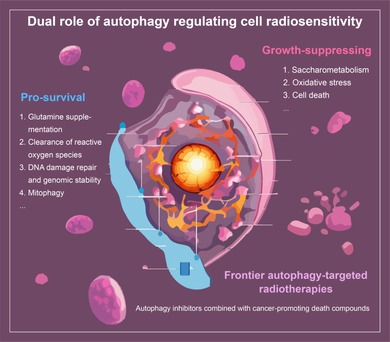

## 1 Introduction

It is written on the front page of the Royal Marsden Cancer Center textbook that there are two ways to cure tumours, a cold scalpel and a searing radiation. Clinical studies have shown that more than 50% of cancer patients need to receive radiotherapy (RT) [1]. However, as the treatment progresses, normal cells will suffer radiation damage and may even die from it before cancer death. Tumour cells with high mutation load may develop radiation resistance, resulting in poor prognosis [2]. In addition, the efficacy of RT is further diminished by the current lack of protection accuracy for radiation injury, the severe side effects of protective medications, and the shortage of sensitising drugs [3]. Therefore, it has been a major scientific problem in radiation medicine to alleviate normal tissue damage and enhance tumour radiosensitivity by identifying new targets and exploring new adjuvant means of RT.

Traditionally, apoptosis constitutes most cases of radiation‐induced cell death. However, in recent years, the differences in cell type, radiation dose, body state, and post‐irradiation time, etc., are endowing ionising radiation (IR) with potentials to trigger various death pathways such as cell necrosis, autophagy, aging, pyroptosis, and ferroptosis [4, 5, 6, 7], which greatly complements the contemporary concepts of biological damage mechanisms of IR. Among them, autophagy, as a rather important way of programmed cell death, is gradually proven to play a significant role in the field of radiobiology. Under a variety of physiological conditions, it secures cell homeostasis by virtue of a recycling mechanism for cell components. During this endogenous transparent system, pyruvate degrades and recycles long‐lived proteins and damaged organelles such as endoplasmic reticulum (ER), Golgi apparatus, and mitochondria through lysosomal/vacuolar processes to sustain cell integrity [8].

Albeit the crucial role of autophagy in cell radiobiological response has been adequately evidenced, the underlying mechanism has not been fully elucidated. Therefore, this review provides an overview of the recent research progress on the autophagy pathways in the regulation of radiosensitivity. Unlike previous similar reviews in the field, this review dissected and integrated for the first time the related pathways of autophagy regulating cell radiosensitivity in recent years. It also analysed the latest developments of autophagy in irradiated normal tissues and tumours, and outlined the frontier RT adjuvant therapies targeting autophagy to regulate tumour radiosensitivity, in an attempt to provide new targets for the development of high‐efficiency and low‐toxicity radiation sensitizers.

## 2 Autophagy: The Process of Cell Recycling

### 2.1 Autophagy: Cyclic Regeneration of Energy and Nutrients in Living Organisms

Autophagy is a highly evolved and conserved physiological process in all eukaryotes. It maintains the delicately balanced cell homeostasis by transporting proteins and organelles that are damaged, deformed, aged, or functionally lost to lysosomes, where these materials are digested and degraded in response to certain abnormal stimuli, such as radiation, starvation, hypoxia, pathogen infection, and oxidative stress. This degradation by autophagy recycles cellular components and eradicates toxic or damaged substances to provide the energy necessary for homeostasis during metabolic stress. Autophagy regulates cell health and longevity through “housekeeping” and protein quality control, thus having a bearing on senescence, immunity, neurodegeneration, and cell death [9]. Studies have reported that the dysfunction of the autophagic process is associated with a wide range of diseases, including cancer, neurodegeneration, metabolic disorders, and infections [10].

**FIGURE 1:**
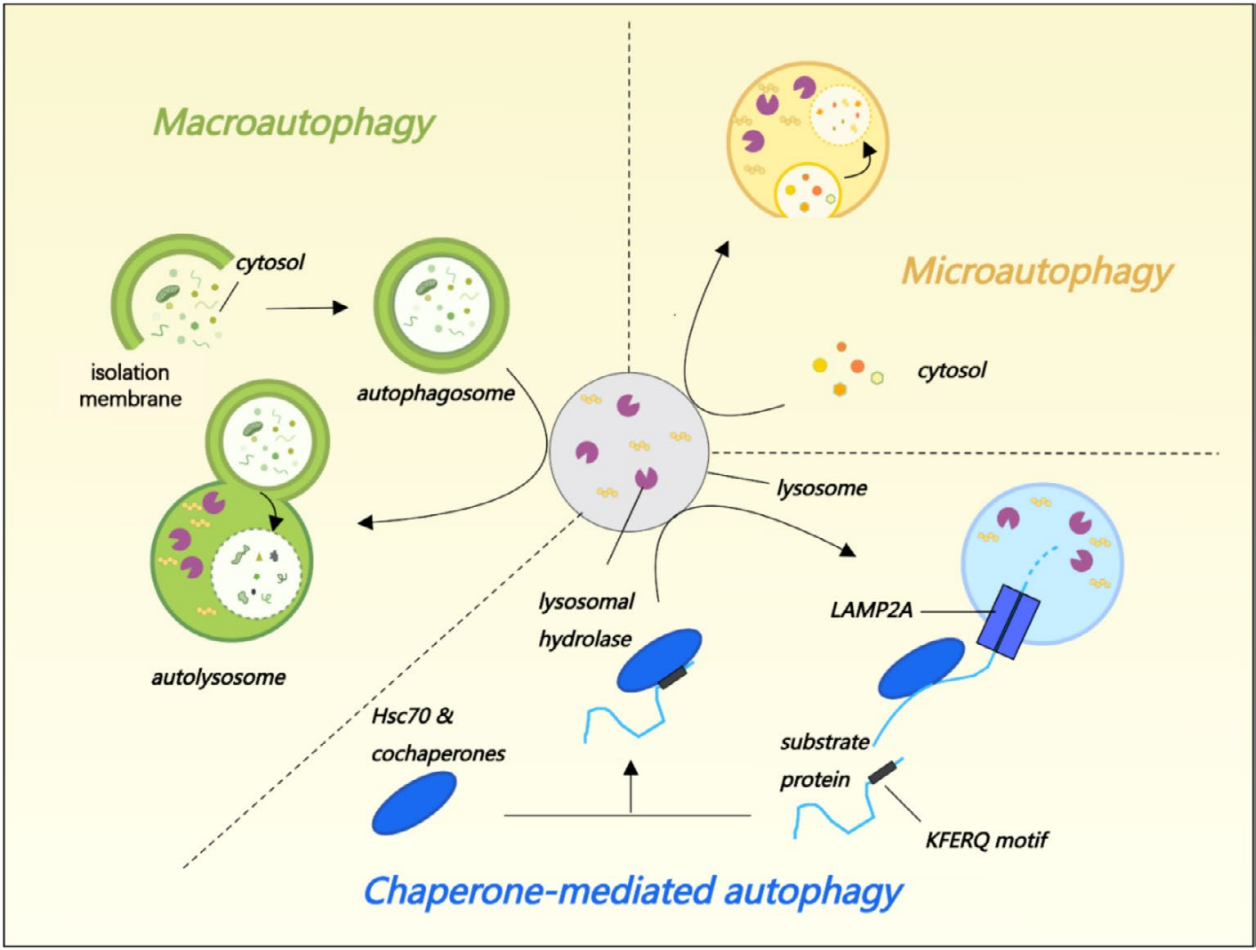
Schematic illustration of different types of autophagy. Cellular autophagy is classified into three distinct types: Macroautophagy, microautophagy, and molecular chaperone‐mediated autophagy (CMA). Macroautophagy: De novo synthesis of autophagosomes is utilised to sequester cargo and subsequently transport it to the lysosomes for degradation. Microautophagy: Invaginations or protrusions of the lysosomal membrane are formed to capture cargo to be degraded within the lysosomes. CMA: Aggregated proteins bind to molecular chaperones and are targeted with a pentapeptide motif KFERQ for degradation by lysosomal proteases in the lysosomal lumen.

Three major types of autophagy have been characterised in accordance with the way in which intracellular substrates enter the lysosome: macroautophagy, microautophagy, and chaperone‐mediated autophagy (CMA) (Figure 1). Macroautophagy is the de novo assembly and maturation of novel double‐membrane vesicles (termed autophagosomes) that expand and approach to sequester cytoplasm in order to efficiently deliver surplus or redundant cellular cargo to the lysosomes for degradation and recycling. During this process, a panel of autophagy‐related (ATG) gene products orchestrates the formation of the autophagosome (the morphological marker of macroautophagy), which later encapsulates cellular cargo and fuses with lysosomes to render the degradation of its contents through the activities of lysosomal hydrolases [11]. In microautophagy, cytoplasmic proteins are directly engulfed by the lysosomes via invagination of the lysosomal membrane or cellular protrusions, and are eventually degraded by lysosomal proteases [12]. Apart from proteins, microautophagy also degrades organelles such as mitochondria, lipid droplets, ER, peroxisomes, and even nuclear fragments [13]. CMA refers to the process in which molecular chaperones recognise cytoplasmic proteins with characteristic pentapeptide motifs (KFERQ sequences), and mediate the binding of proteins to monomers of the CMA substrate receptor, namely lysosomal‐associated membrane protein 2A (LAMP2A), and then are degraded by lysosomes. During CMA, complexes containing hsc70 are involved in protein recognition and unfolding [14, 15].

Meanwhile, in terms of selectivity, autophagy is additionally classified into non‐selective autophagy and selective autophagy. Most of the former occurs in the absence of nutrients, aiming to non‐selectively catabolise cytoplasmic components into structural units, such as amino acids, so as to support protein synthesis to adapt to hunger and the energy supply required for cell survival. When nutrient‐rich, the latter occurs at a low level, mediating the overall turnover of cytoplasmic materials [16]. Selective autophagy encompasses mitophagy, lipophagy, peroxophagy, ribophagy and so on [12, 17, 18]. This selectivity is facilitated by autophagy cargo receptors (ACRs), including p62 and p53, which bind to the very cargo that has been targeted for degradation via ubiquitin‐dependent or ubiquitin‐independent processes [19]. Selective autophagy can be initiated continuously to maintain intracellular homeostasis, whose necessary pathways can be activated by specific stimuli to manage specific stressors [17]. Selective autophagy also plays a role in a variety of disease pathologies, including atherosclerosis, neurodegeneration, and cancer [20].

Macroautophagy (hereafter referred as autophagy) will be mainly addressed in this review, on account of its importance as the principal form of autophagy that directly degrades a substantial amount of cellular contents through autophagosomes, whereas other forms of autophagy have relatively limited scopes and mechanisms.

### 2.2 Autophagy Cell Signal Transduction Pathway

**FIGURE 2:**
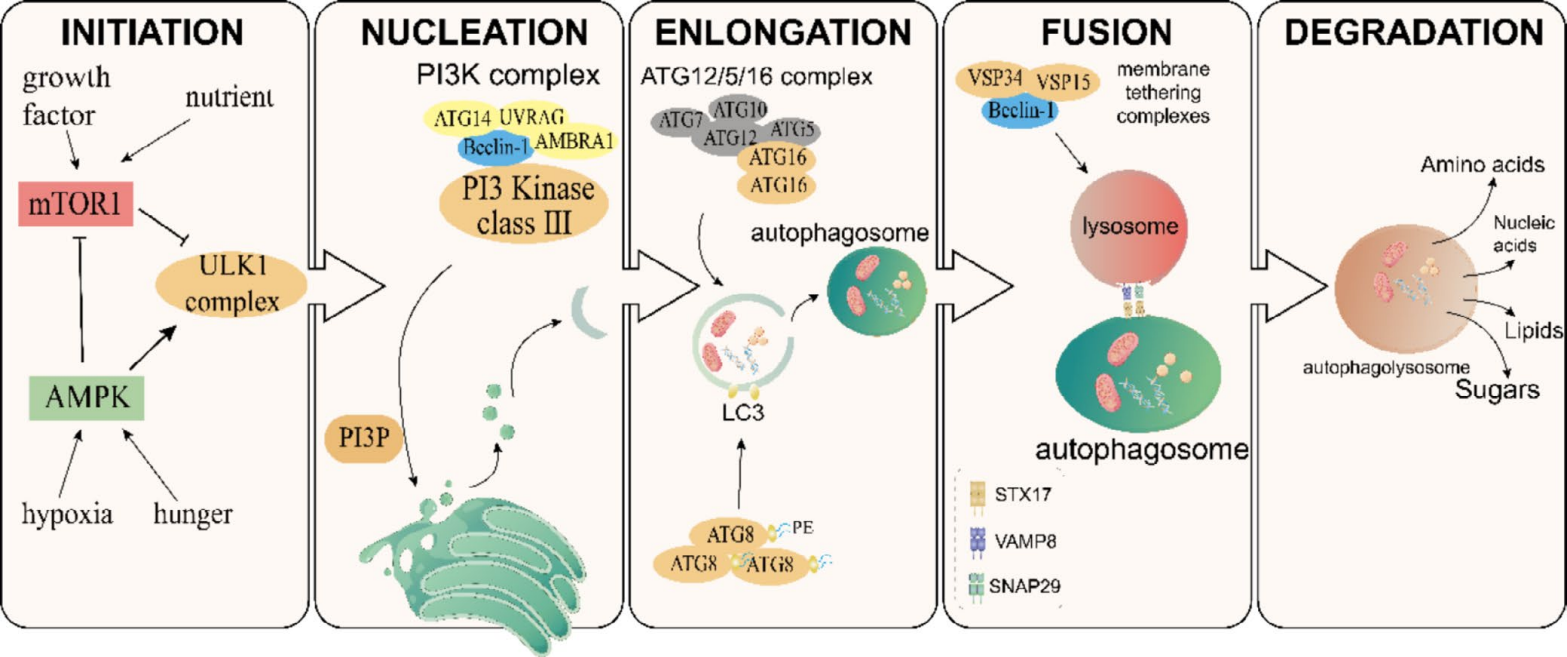
Multiple steps involved in the autophagy pathway. The autophagy pathway consists of five steps. Initiation: The Unc‐51 like kinase 1 (ULK1) complex integrates signals from regulators of the mammalian target of rapamycin complex 1 (mTORC1) and AMP‐activated protein kinase (AMPK) to activate autophagy. Nucleation: Class III phosphatidylinositol‐3‐kinase (PI3K class 3) mediates the formation of phagophores. Elongation: The ATG12‐ATG5‐ATG16 complex and the ATG8/LC3 system coordinate to secure phagophore elongation. Fusion: Docking and fusion between autophagosomes and lysosomes are regulated by Rab GTPases, membrane‐tethering complexes, and soluble N‐ethylmaleimide‐sensitive factor attachment protein receptors (SNARE). Degradation: Lysosomal degradation and recycling of autophagic cargo.

In eukaryotes, the core mechanism of autophagy has a highly conserved core mechanism that incorporates five main steps: initiation, nucleation, elongation, fusion, and degradation (Figure 2). Among the approximately 20 related proteins involved, specific core ATG proteins are respectively highlighted in the above steps. The current understanding of the major autophagic process is summarised as follows.

Step 1: Initiation. The Unc‐51 like kinase 1 (ULK1) complex regulates the formation of autophagic vesicles (phagophore). Usually under normal cell conditions, this complex stays inactive due to the phosphorylation by the mammalian target of rapamycin complex 1 (mTORC1). However, when exposed to autophagy‐inducing stimuli such as nutritional starvation, hypoxia, etc., AMP‐activated protein kinase (AMPK) will be activated by a low‐energy state to further activate the ULK1 complex with serine–threonine kinase activity, thus directly initiating autophagy. At the same time, AMPK lifts the inhibition of mTORC1 on the ULK1 complex by inhibiting the activity of mTORC1, thereafter activating autophagy in an indirect manner [21]. Overall, the ULK1 complex integrates signals from regulators of both nutrient (mTORC1) and energy stress (AMPK) in the cells [22].

Step 2: Nucleation. This process assists the proper localisation of other autophagy proteins to form phagophores, and is primarily mediated by class III phosphatidylinositol‐3‐kinase (PI3K class 3). Beclin‐1 is an intriguing component of the PI3K complex. Phosphorylated Beclin‐1 is able to recruit ATG14, AMBRA‐1, and UVRAG to the PI3K complex, allowing for the latter to generate phosphatidylinositol 3‐phosphate (PI3P) on the membrane to function in the autophagosome nucleation [22].

Step 3: Elongation. There are two ubiquitin‐like (UBL) conjugation systems governing the elongation of phagophores [23]. The first system involves the formation of the ATG12‐ATG5‐ATG16 complex. During this process, the UBL protein ATG12 is covalently conjugated to ATG5 in a manner dependent on ATG7 and ATG10. Then, ATG16 binds to ATG5 noncovalently and dimerises to form a larger complex that binds to the phagophore to promote the maturation and stability of the autophagosome. The second UBL system is the ATG8/LC3 system, during which ATG8/LC3 forms a covalent connection with a lipid chaperone phosphatidylethanolamine (PE) to trigger the indispensable formation of autophagosomes. After that, a series of protein‐to‐lipid conjugation cascades attach an LC3 family member protein to the membrane lipid of autophagic vesicles, which identifies the vesicle as a phagophore and facilitates the receipt of cargos [24]. Basically, the ATG12‐ATG5‐ATG16 complex focuses more on the maturation and stability of autophagosomes, while the ATG8/LC3 system is more involved in the formation and expansion of autophagosomes. These two systems secure the smooth development of the autophagic process by playing their respective roles in different stages of autophagosomes.

Step 4: Fusion. This is a step regulated by Rab GTPases, membrane‐tethering complexes (such as HOPS complex and VPS genes) and soluble N‐ethylmaleimide‐sensitive factor attachment protein receptors (SNARE) [25]. Following the elongation of phagophores, cargos are trapped either in bulk or in a selective manner and are encapsulated by autophagosomes to be later fused with lysosomes to form autolysosomes [26]. The transport of phagophores comprises budding, transporting, tethering, and fusion of vesicles. Notably, the HOPS complex, as an important tethering factor, is capable of making autophagic vesicles and the target membrane closer to each other and promote fusion at the initial stage of contact, so as to mediate the initial connection between vesicles. SNARE proteins, such as STX17 on the autophagosome membrane and VAMP8 and SNAP29 on the lysosomes, can provide specificity for cargo transport by recognising each other, thereby ensuring the orderly progression of the entire process [27]. Additionally, the largest subfamily Rab protein, such as RAB7 in the small molecule GTP‐binding protein family, acts as a molecular switch for vesicle transport and promotes fusion with the assistance of the LC3 family proteins [28].

Step 5: Degradation. Eventually, these aggregated proteins or dysfunctional organelles are completely degraded by cathepsins and lysosomal hydrolases in a pH‐dependent manner in autophagic lysosomes. After degradation, metabolites are released into the cytoplasm and recycled in metabolism to synthesise new materials that are essential for cell viability, such as functional proteins, enzymes, and ATP.

## 3 Radiosensitivity

### 3.1 Foundations in Radiobiology

Nowadays, the putative definition of radiosensitivity refers to the degree of morphological and functional changes in organisms, cells, tissues or organs after receiving IR, as well as the rate of injury, death or other adverse effects [29]. The effect of radiation on cells depends on the “4Rs” *principle in radiobiology, namely repair, reoxygenation, redistribution and regeneration* [30].

Radiosensitivity varies significantly among cells, tissues, and organs in different developmental and physiological states of the human body. At the level of tissues and organs, radiosensitivity is higher in the early stage of multicellular organisms (e.g., all organs of the fetus) and cells with vigorous cell division (e.g., germ cells, haematopoietic tissues, intestinal epithelium, etc.), while it is lower in adult tissues with slow cell division such as the brain, muscle, liver, and mature blood cells (except lymphocytes) [31, 32, 33]. Besides, it is well worth noting that the radiosensitivity of tumours of different pathological types is quite different as well, which leads to the consequently uneven therapeutic efficacy of RT among tumours. Tumour cells with high differentiation such as seminoma and malignant lymphoma are insensitive to radiation therapy, while tumour cells with low differentiation such as adenocarcinoma and glioblastoma are more sensitive to radiation because their cell cycle is more active and they are more susceptible to radiation damage [34, 35].

Based on a myriad of radiopathological data, the Common Terminology Criteria for Adverse Events (CTCAE) [36] and the Radiation Therapy Oncology Group (RTOG) scale [37] have classified the degree of radiation‐induced response in each organ from light to heavy to provide the most reliable reference for clinical quantification of radiosensitivity [38]. A variety of indicators can be adopted to measure the radiosensitivity. In most cases, the survival rate is expressed by the clone formation rate, especially SF2 (i.e., the survival rate after 2Gy irradiation), which is one of the most effective parameters to quantify the radiosensitivity of cells [39].

### 3.2 DNA Damage Response

The cellular radiosensitivity of tumours is the pivotal point to the preferable efficacy of RT. However, in both tumour cells and normal tissues, the radiosensitivity is comprehensively regulated by multiple factors, including DNA damage repair ability, cell cycle progression, and intrinsic autophagy level [40].

For example, cell DNA damage repair is a key factor in radiosensitivity. IR elicits a series of structural changes in biological macromolecules through direct and indirect effects, with its central target at DNA molecule damage [41], which plays a paramount role in the reaction to radiation exposure, steering the subsequent cascade of DNA repair response signalling pathways to regulate the arrest of the cancer cell cycle and determine cell fate [42]. DNA damage includes single‐strand breaks (SSBs), double‐strand breaks (DSBs), base damages, and cross‐linking, etc. [42]. Among them, DSBs are the most severe form of DNA damage response after radiation and are a prior mechanism to kill cancers for RT [43], due to their probable loss of large chromosomal regions in the absence of timely repair. There are two main pathways for DNA DSB repair, the first of which is the error‐prone non‐homologous end joining (NHEJ) pathway, which is activated through the cell cycle; the other one is called the homologous recombination repair (HRR) pathway, which occurs during the late S and G2 phases and is considered an error‐free repair mechanism of DSBs, during which the homologous sequence on the sister chromatid works as the precise template to fill in the DSB gaps in order to restore the original DNA sequence. Compared with HRR, NHEJ is given the probability of short insertions/deletions of genomic DNA, and is then regarded as an error‐prone repair mechanism. During NHEJ, 53BP1 is vital in protecting broken DNA ends from extensive resection in the XRCC4‐dependent pathway, and is accordingly referred to as the core protein of NHEJ [44]. Patients with head and neck cancer who had low 53BP1 expression levels and were treated with radiotherapy, as demonstrated in a clinical study by Cirauqui et al., experienced a higher complete response rate and longer survival times compared to those with high 53BP1 expression levels [45]. Meanwhile, it has been demonstrated that Rad51, the critical protein in the HRR pathway, and Ku70, Ku80, the critical proteins in the NHEJ pathway, are of unyielding significance in DSB repair induced by RT [46, 47]. Balbous A et al. proved a radiosensitizing effect of RAD51 inhibition in glioblastoma stem‐like cells [48]. The Ku80‐Ku70 heterodimer is a component of the DNA‐dependent protein kinase (DNA‐PK), which acts as the molecular sensor for DSB. Multiple studies have shown that inhibiting DNA‐PK can enhance cellular sensitivity to IR and DNA‐damaging agents [49, 50, 51]. Overall, increased expression of DSB repair proteins will cultivate radioresistance [52]. And radiosensitivity can be concisely described as the cellular capacity to perform specifically DNA DSB repair [53].

### 3.3 Autophagy‐Radiosensitivity Interplay

Concurrently, studies also indicate that radiosensitivity is related to the cell cycle and can be regulated by autophagy. For instance, it was reported by Liang et al. [54] that the autophagy inhibitor 3‐methyladenine (3MA) could mitigate the S‐phase delay induced by IR, thereby exerting an enhancing effect on the radiosensitivity of the human ovarian carcinoma cell line SKOV3.

Hence, another distinctive way to regulate radiosensitivity is autophagy. The aforementioned cytoprotective function of autophagy reflects the capacity of cells to eliminate toxic species such as free radicals, damaged proteins, and unwanted organelles to generate energy and metabolic precursors. For example, an excess of free radicals within the cellular environment can be detected by lysosomes and then eliminated via activated calcium ion channels [55]. Also, activation of autophagy can protect vascular endothelial cells and T cells from IR damage. However, when such a protective effect becomes excessive, the degradation of cell organelles and key proteins will exceed the body's compensatory amount, resulting in autophagic cell death, or more specifically, type II programmed cell death [56]. In this way, present academic discussion on the relationship between autophagy and radiosensitivity is mainly oriented to two perspectives. Firstly, different radiation treatments regulate cell survival rates by perturbing autophagy to alter intracellular toxic substances. Secondly, radiation‐induced autophagy regulates cell radiosensitivity as an additional cell death pathway triggering downstream reactions [57].

## 4 Research Status of Autophagy in Regulating Cell Radiosensitivity

### 4.1 Dual Role of Autophagy in the Regulation of Tumour Cells

The impact of autophagy under any physiological or pathological conditions seems to be highly context‐dependent. In different environments and stages of tumour progression, autophagy can play a neutral, tumour‐inhibiting, or tumour‐promoting role depending on the availability of nutrients, the tension of the microenvironment, pathogenic properties, and the activation of immune mechanisms [58].

Autophagy exerts a multifaceted, dichotomous influence on the development of cancer. On the one hand, it has been demonstrated to promote cancer through several mechanisms. It maintains cancer stem cells (CSCs) [59], which are crucial for tumour initiation and recurrence, and regulates cancer cell invasion and metastasis by modulating enzymes that degrade the extracellular matrix [60]. Autophagy also provides the minimal energy for cancer cells to survive under starvation and stress through self‐digestion [10]. Additionally, it supplies metabolites necessary for cancer cell maintenance and resistance to apoptosis or necrosis, such as anti‐apoptotic protein Bcl‐2 and TNF‐α, while protecting cancer cells from immune responses [61]. Moreover, autophagy has been observed to regulate the cancer microenvironment, thereby supporting metastasis and resistance to stressors in advanced stages of tumourigenesis [62].

Conversely, autophagy has the capacity to impede the initiation of tumours and to eradicate cancer cells during tumour progression. It removes abnormal cells and organelles, reducing stress on tumour‐related genes and preventing genomic instability and malignant transformation [63]. As a central regulator of the inflammasome, autophagy can inhibit inflammation and reduce the risk of cancer [64]. The autophagy cargo receptor p62 is a cohesion protein in various cancer‐related signalling pathways. The abnormal accumulation of p62 could be prevented by autophagy to inhibit tumours caused by oxidative stress and tissue damage [65]. Autophagy also inhibits the epithelial‐mesenchymal transition (EMT) of cancer cells by degrading the p62 protein and transporter TWIST1, finally inhibiting tumour cell metastasis [66].

Tumour progression is classified into distinct stages, including carcinoma in situ, localised tumour growth, regional lymph node metastasis, and distant metastasis. Each stage exhibits unique biological characteristics, tumour microenvironment features, and therapeutic responses [67]. For instance, in colorectal cancer, procollagen‐lysine, 2‐oxoglutarate 5‐dioxygenase 3 (PLOD3) expression demonstrates a positive correlation with tumour stage, lymph node involvement, and distant metastasis, with elevated expression levels being associated with unfavourable clinical outcomes [68]. This observation suggests that dynamic alterations in intracellular molecular mechanisms and signalling pathways occur throughout cancer progression, ultimately influencing tumour aggressiveness and therapeutic sensitivity.

Emerging evidence is suggesting that the functional consequences of autophagy are stage‐dependent during cancer progression. During early tumorigenesis, autophagy maintains cellular homeostasis through quality control mechanisms, thereby suppressing malignant transformation. While in advanced malignancies, autophagy may promote tumour cell survival by conferring resistance to therapeutic interventions, including radiotherapy [69].

It is also noteworthy that the function of autophagy in enhancing or reducing the radiosensitivity of tumour cells is influenced by cancer types and tumour microenvironment. For instance, in non‐small cell lung cancer (NSCLC), studies have indicated that the combination treatment of NSCLC cells with rapamycin and histone deacetylase inhibitors promotes autophagy while inhibiting DNA damage repair, thereby enhancing cellular radiosensitivity [70]. The study by Wang J et al. [71] demonstrates that inhibition of the AURKA‐CXCL5 axis induces autophagic cell death and promotes radiosensitivity in NSCLC. Additionally, research suggests that autophagy plays a role in regulating the radiosensitivity of NSCLC cells under hypoxic conditions, enhancing radioresistance by modulating the level of reactive oxygen species (ROS).

In breast cancer, studies have demonstrated that selective inhibition of PLK1 can reduce autophagy levels, thereby enhancing their sensitivity to RT [72]. Similarly, miR‐26b inhibits autophagy in breast cancer cells by targeting the damage regulated autophagy modulator 1 (DRAM1) mRNA, and this inhibitory effect can augment the cancer cells' radiosensitivity [73]. In contrast, RT‐induced autophagy in breast cancer cells was also shown to be associated with ferroptosis. Enhanced autophagy leads to iron accumulation, which in turn triggers oxidative stress and DNA damage, ultimately inducing cell death [67].

Glioma is a highly malignant brain tumour characterised by its low sensitivity to RT. Studies have indicated that autophagy plays a significant role in glioma cells' radiosensitivity. Research findings suggest that Lidoflazine enhances radiosensitivity by inhibiting nuclear receptor binding factor 2 (NRBF2)‐mediated autophagy [74]. However, there is also a subsequent study revealing that transient exposure of glioma stem cells (GSCs) to rapamycin induces cellular differentiation and markedly increases radiosensitivity through the activation of autophagy [75].

In summation, autophagy plays a dual role in tumour control. It is urgently needed for autophagy‐targeted tumour therapies to develop strategies to control the regulatory direction. This dynamics correlates with cancer type, stage, metabolic requirements, and is inevitably relevant to the microenvironment tension, immune mechanism, and autophagy‐regulating transcription factors [58]. Such a dual role of autophagy on tumour growth is visibly reflected in the regulation of radiosensitivity.

### 4.2 The Mechanism of Autophagy Enhancing Cell Radiosensitivity

**FIGURE 3:**
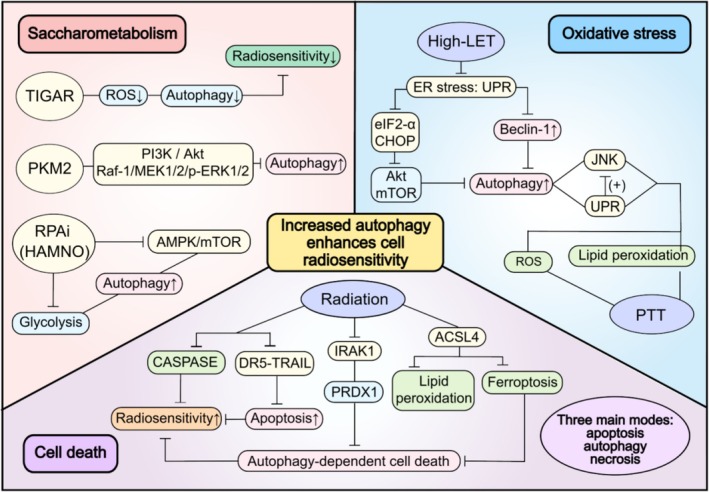
The mechanism of autophagy enhancing cell radiosensitivity. Saccharometabolism: TP53‐induced glycolysis and apoptosis regulator (TIGAR) inhibits autophagy by scavenging ROS to decrease radiosensitivity. Silencing pyruvate kinase M2 (PKM2) expression enhances autophagy and increases radiosensitivity via PI3K/Akt and Raf‐1/MEK1/2/p‐ERK1/2 pathways. RPAi (HAMNO) suppresses glycolysis to enhance autophagy, thereby increasing radiosensitivity. Oxidative stress: High linear energy transfer (LET) radiation induces autophagy through inactivation of Akt–mTOR and upregulation of Beclin‐1 expression in the unfolded protein response (UPR) pathway, so as to enhance radiosensitivity. JNK and UPR activation under photothermal therapy (PTT) treatment also induce autophagy and strengthen radiosensitivity. Cell death: Autophagy‐dependent cell death that increases radiosensitivity can be triggered by the death receptor 5 (DR5) pathway with the regulation of apoptosis and caspase levels, and can be prevented by the interleukin‐1 receptor‐associated kinase 1 (IRAK1) through reduced ubiquitination of peroxiredoxin 1 (PRDX1). Under radiation, lipid peroxidation and ferroptosis were triggered by the expression of ROS and long‐chain acyl‐CoA synthetase 4 (ACSL4), in which way cell radiosensitivity is promoted.

Our study delved into the intricate relationship between autophagy and cell radiosensitivity. The insights into the radiosensitising mechanisms of autophagy during RT have been summarised in Figure 3.

#### 4.2.1 Saccharometabolism

Reprogramming of cellular metabolism plays an essential role in tumour initiation, progression, and RT resistance. Under aerobic conditions, tumour cells will increase glycolysis rather than oxidative phosphorylation to meet their energy needs, that is, the Warburg effect. This metabolic phenotype difference then becomes the basis for autophagy‐regulated radiosensitivity of tumours without compromising normal tissues [76]. Basal autophagy levels are low in normal situations, but are profoundly up‐regulated and more autophagy‐dependent than normal cells in response to specific stimuli such as glucose deprivation [77]. Therefore, in‐depth research into the mechanisms by which glycolysis affects tumour radiosensitivity via autophagy is of great significance for enhancing the efficacy of RT.

TP53‐induced glycolysis and apoptosis regulator (TIGAR) functions as fructose‐2,6‐bisphosphatase (FBPase‐2), which inhibits glycolysis and activates the pentose phosphate pathway (PPP) under oxidative stress. In the study of human parotid gland fibroblasts Hs 917, T cells, Tai et al. [78] observed that TIGAR overexpression may inhibit autophagic activity by scavenging intracellular ROS, thereby decreasing the radiosensitivity of salivary gland fibroblasts Hs 917.

Aerobic glycolysis, as the metabolic hallmark of cancer, has been proven to be associated with radioresistance in NSCLC. Pyruvate kinase M2 (PKM2) is expressed exclusively in cancers as a key regulator of glycolysis. Meng et al. [79] have explicated that silencing PKM2 expression enhanced IR‐induced apoptosis and autophagy in vitro and in vivo through the PI3K/Akt and Raf‐1/MEK1/2/p‐ERK1/2 pathways, thereby intensifying the radiosensitivity of NSCLC cell lines and transplanted tumours, which may redound to the design of innovative therapies for NSCLC targeting autophagy.

Feng et al. [80] have shown that replication protein A inhibitor (RPAi) facilitated the ATG gene transcription by activating AMPK/mTOR, inducing autophagic flux and glycolysis, thereby inducing autophagy at multiple levels to improve the radiosensitivity of nasopharyngeal carcinoma (NPC) cell lines. They identified a potent RPAi (HAMNO), which was closely related to the enhanced radiosensitivity and the inhibited proliferation of NPC cell lines in vivo and in vitro. Furthermore, they also proposed that HAMNO induced autophagy by suppressing glycolytic function and activating ATG genes, so as to enhance the radiosensitivity of NPC cell lines.

In brief, glycolysis creates a reductive chemical environment related to the development of radioresistance in cancer cells. The inhibition of glycolysis enhances the autophagy level of cells and effectively upregulates the radiosensitivity of tumour cells, thus contributing to significant therapeutic gains in combination with RT.

#### 4.2.2 Oxidative Stress

Recent studies indicate that under conditions of cellular oxidative stress, radiation can trigger lipid peroxidation, activate multiple radiation response pathways, and enhance cellular radiosensitivity to promote autophagy by damaging DNA, the plasma membrane, and organelles. Deville et al. [81] have studied the head and neck squamous cell carcinoma (HNSCC) and found that the inhibition of Keap1 (a substrate adaptor protein that binds substrates to the E3‐ubiquitin ligase complex) could affect DNA damage repair through autophagy induction, thereby enhancing the radiosensitivity of HNSCC cells.

In the study of irradiated KHOS/NP (a human osteosarcoma cell line derived from the HOS cell line through transformation using Kirsten murine sarcoma virus) and MG63 osteosarcoma cells, Oh et al. [82] clarified the molecular mechanism of high linear energy transfer (LET) radiation inducing autophagy through ER stress to enhance radiosensitivity. They proposed that Akt–mTOR is located downstream of the unfolded protein response (UPR) pathway, with high‐LET carbon ions activating the UPR. The latter induces the phosphorylation of eIF2α and C/EBP‐homologous protein (CHOP), which consequently inhibits Akt and mTOR activation by blocking Akt phosphorylation, leading to autophagy. They also observed that high‐LET neutron exposure increased Beclin‐1 expression during the UPR, thereby enhancing autophagy. Overall, their results demonstrated that autophagy is crucial in radiosensitivity, cell survival, and cellular resistance against high‐LET neutron radiation, and neutrons can induce autophagy by activating ER stress to enhance the radiosensitivity of cells.

Mishra et al. [83] first elucidated the autophagy‐dependent radiosensitising mechanism of stress responses such as lipid peroxidation induced by JNK activation and UPR activation under photothermal therapy (PTT) treatment. The results indicated that the severe oxidative damage of lipid after PTT treatment might be one of the culprits of cell damage after PTT and might be mediated by the increase of ROS. JNK activation was observed to be triggered by oxidised lipids, increased ROS, and UPR signalling in PTT‐treated cells. Such enhanced JNK activation then triggered the induction of autophagy. Moreover, the coexistence of swollen endoplasmic reticulum and autophagic bodies after PTT suggested possible coordination between UPR and autophagy pathways. In sum, marked induction of pJNK, UPR pathway, increased ROS, and lipid peroxidation in PTT‐treated cells disrupted the intracellular homeostasis and provided new evidence for autophagy‐mediated PTT as an independent RT method.

Collectively, autophagy induced under oxidative stress is a critical mechanism for augmenting cellular radiosensitivity. Emerging evidence is suggesting that radiation also modulates autophagy by inducing accumulation of ceramide, ROS, and Ca^2+^, etc., thereby activating signalling pathways that lead to damage of extranuclear targets such as plasma membrane, mitochondria, and ER, rather than solely through the direct or indirect DNA damage and the activation of DNA damage repair signalling pathways as traditionally perceived to be involved in the regulation of autophagy.

#### 4.2.3 Cell Death

Lately, cell death has been proposed as a principal mechanism of radiosensitivity regulation, of which apoptosis, autophagy‐dependent cell death, and necrosis are considered the main modes initiated under radiation [84]. It has been reported that vitamin D and vitamin D analogs such as EB 1089 can enhance the response to radiation in breast cancer through the promotion of a cytotoxic form of autophagy [85, 86]. Hence, autophagy is expected to have the potential to promote cell killing in radiation responses.

Zhang et al. [87] demonstrated that autophagy can increase radiosensitivity by promoting cell death through the death receptor 5 (DR5) pathway. Results revealed that autophagy inducers affected DR5 transcription in the irradiated human glioma cell line U251 via histone H3 trimethylation (H3K27me3) at its promoter region, likely mediated by altered activity of histone methyltransferases or demethylases. It was further pointed out that autophagy could decrease the survival rate of radiation‐tolerant cells by increasing the apoptosis and caspase levels of U251 cells after irradiation. Moreover, TNF‐related apoptosis‐inducing ligand (TRAIL) was found to recruit autophagic proteins into the death pathway, thereby triggering apoptosis.

However, there are also studies implying that autophagy can trigger pro‐survival mechanisms and resist apoptosis. For example, autophagy can reduce apoptosis by removing damaged or degenerated subcellular components and harmful pathogens in a selective manner, while maintaining genomic integrity under various interferences such as metabolic stress, drug toxicity, or radiation damage. It can also decompose metabolic organelles and macromolecules to provide nutrients and energy sources for starved cells [88].

Other cell death mechanisms are also identified to play an important role in regulating cell radiosensitivity. For example, Li et al. [89] showed that radiation can reduce the ubiquitination of peroxiredoxin 1 (PRDX1) in glioma cells by inducing the expression of interleukin‐1 receptor associated kinase 1 (IRAK1), thereby inhibiting autophagic cell death and promoting radioresistance. In addition, IR was found to induce the expression of ROS and long‐chain acyl‐CoA synthetase 4 (ACSL4), a lipid metabolism enzyme required for ferroptosis, and was accordingly responsible for the increased lipid peroxidation and ferroptosis [7]. This finding highlighted the enhancement of radioresistance by inhibiting ferroptosis. Excessive autophagy activation has gradually been confirmed to be a radiosensitivity‐enhancing mechanism that mediates ferroptosis [90].

The cell death pathway is vitally a mechanism underlying the autophagy‐mediated cell radiosensitization. Numerous studies have noted a synergistic effect between multiple drugs and radiation, and the mechanism is to promote autophagic cell death rather than other cell death pathways [91]. Therefore, cell death, especially autophagy‐dependent cell death, might present a favourable scenario for enhancing the efficacy of RT and developing RT sensitizers.

### 4.3 The Mechanisms Underlying the Autophagy‐Induced Tumour Radioresistance

**FIGURE 4:**
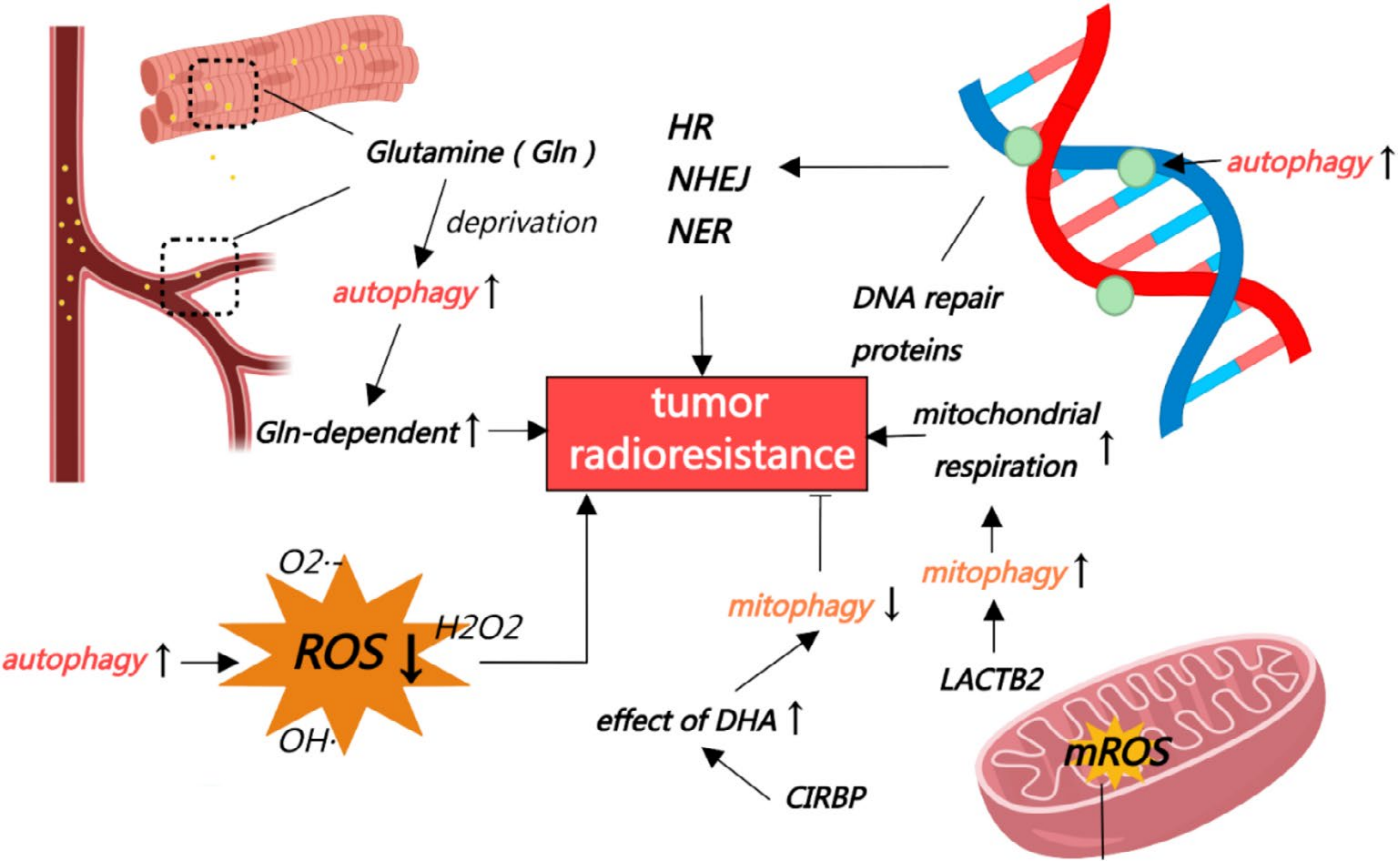
The mechanisms of autophagy‐induced tumour radioresistance. Autophagy activation under glutamine‐deficient conditions increases radioresistance of Gln‐dependent PCa cells. Under hypoxic conditions, autophagy enhances radioresistance of tumour cells by reducing ROS. DNA damage‐induced autophagy affects homologous recombination (HR), non‐homologous end‐joining (NHEJ) and nucleotide excision repair (NER) by regulating DNA repair proteins, thereby enhancing tumour cell radioresistance. Mitophagy activated byβ‐lactamase‐like protein 2 (LACTB2) can foster radioresistance by enhancing mitochondrial respiration in peri‐tumour cells. Dihydroartemisinin (DHA) reduces radiation‐induced mitophagy and decreases tumour cell radioresistance through the inhibition of cold‐inducible RNA‐binding protein (CRIBP).

The investigation into the mechanisms of autophagy inducing cell radioresistance has been encapsulated in Figure 4.

#### 4.3.1 Autophagy Promotes Glutamine Supplementation in Tumour Cells

Glutamine (Gln) is the most abundant circulating amino acid in blood and muscle, and is largely consumed in many tumours including pancreatic cancer, ovarian cancer, and breast cancer. These proliferating tumour cells mainly use Gln as their primary energy source to support the TCA cycle.

Evidence has highlighted autophagy's role in Gln‐mediated cancer metabolic regulation. It was found by Strohecker et al. [92] that autophagy loss could impair lung cancer cell respiration and survival via Gln‐dependent mechanisms. Mukha et al. [93] further reported that a combination of Gln metabolism blockade and autophagy inhibition might be a promising strategy for prostate cancer (PCa) radiosensitisation. In response to Gln deprivation, PCa cells with acquired radioresistance can activate ATG5‐mediated autophagy and trigger metabolic reprogramming as a survival mechanism to resist radiation damage and overcome nutrient stress. Mukha et al. demonstrated for the first time that the inhibition of autophagy in these PCa cells significantly increased the radiosensitising effect of Gln deprivation. Therefore, inhibition of autophagy in the context of blocking Gln metabolism enhances the radiosensitisation of PCa cells.

#### 4.3.2 Autophagy Accelerates the Clearance of ROS in Tumour Tissues Under Hypoxia Condition

ROS is a family of highly reactive molecules which includes free oxygen radicals, such as superoxide anion (O_2_^·‐^), hydroxyl radical (OH^·^) and non‐radical oxygen derivatives like the stable hydrogen peroxide (H_2_O_2_). Radiation promotes the accumulation of ROS, and the latter plays a direct role by directly modifying biomolecules such as proteins, lipids, and nucleic acids to elicit cell dysfunction and energy imbalance. The production of toxic ROS is one of the most important mechanisms for the effect of RT.

Current studies have addressed that autophagy scavenges ROS and alleviates oxidative stress in cells. Feng et al. [94] demonstrated that hypoxia preconditioning attenuated radiation‐induced ROS generation in MG‐63 osteosarcoma cells via autophagy activation‐mediated clearance, conferring enhanced radioresistance. Chen et al. [95] reported that autophagy enhanced the radioresistance of NSCLC by regulating ROS levels under hypoxia conditions. Compared with wild‐type A549 cells, they found that autophagy significantly reduced ROS levels and reduced the radiosensitivity of lung cancer cells at the same radiation dose. LC3 siRNA treatment was observed to reduce autophagy, increase ROS stress, and enhance cell radiosensitivity. After hypoxia stress, the number of mitochondria was reduced, while the cellular ROS level was enhanced. These results revealed a possibility that cell radioresistance can be enhanced by increasing the induction of autophagy in response to hypoxia stress. During hypoxic treatment, autophagy may raise cell radioresistance by reducing ROS.

#### 4.3.3 Autophagy Enhances DNA Damage Repair and Genomic Stability in Tumour Cells

DNA is a highly sensitive target molecule to IR. Irradiated cells tend to trigger a DNA damage repair response accompanied by autophagy regulation. Studies have analysed lysosomal nuclease Dnase2a‐deficient mice and found that damaged DNA is usually exported from the nucleus and then cleared by lysosomes through autophagy [96]. Autophagy activation can attenuate the degree of DNA damage induced by endogenous or exogenous factors, thereby reducing the cytotoxicity of damaged DNA and contributing to cell survival.

In glioblastoma research, inhibition of autophagy (e.g., Bafilomycin A1) was shown to significantly impair DSB resolution, demonstrating its regulatory role in modulating DNA repair machinery to orchestrate tumour cell damage response [97]. Zhang Dan [98] found that autophagy induced by DNA damage directly affects homologous recombination (HR), NHEJ, and nucleotide excision repair (NER) by maintaining a balance between the synthesis, stabilisation, and degradation of DNA repair proteins in the degradation pathway to promote DNA repair. Therefore, down‐regulation of autophagy to inhibit the DNA repair pathway might be an effective method to inhibit radiosensitisation and improve anticancer efficacy.

#### 4.3.4 Mitophagy Can Improve the Mitochondrial Function of Tumour Cells

Mitophagy is an important selective autophagy, which refers to the clearance and renewal of damaged mitochondria to maintain mitochondrial quality and the homeostasis of the intracellular environment. Several studies have suggested that mitophagy contributes to tumour growth and radioresistance [99, 100].

The regulation of mitophagy on tumour radiosensitivity branches into two aspects, based on the inducement of mitophagy. On the one hand, when mitophagy is induced by mitochondrial ROS (mROS), it can promote the sensitivity of tumour cells to IR [101]. Similarly, ROS‐induced autophagy was also observed to initiate the sensitisation of cancer cells to IR in the breast cancer cell line MCF‐7 and the cervical cancer cell line HeLa [100]. On the other hand, in some cases, inhibition of mitophagy promotes the radiosensitivity of tumour cells. Studies by Chen et al. [102] have shown that β‐lactamase‐like protein 2 (LACTB2) renders radioresistance by activating mitophagy in nasopharyngeal carcinoma. In nasopharyngeal carcinoma resistant cells, activated mitophagy can cause enhanced mitochondrial respiration, which is a key factor in mediating nasopharyngeal carcinoma RT resistance. When mitophagy is absent or inhibited, the radiosensitivity of cells to radiation may be enhanced. In addition, the study of dihydroartemisinin (DHA) by Wu et al. [103] also found that mitophagy can enhance the radioresistance of cells. They observed that cold‐inducible RNA binding protein (CIRBP) was highly expressed in A549R cells. The knockdown of CIRBP increased the effect of DHA, reduced mitophagy and radioresistance, and inhibited the mitophagy‐related PINK1/Parkin pathway. Therefore, CIRBP might be a potential target of DHA‐regulating mitophagy. DHA might reduce radiation‐induced mitophagy and radioresistance of lung cancer A549 cells via CIRBP inhibition.

In summary, mitophagy contributes to the clearance and renewal of damaged mitochondria, protects cells from stress, and enhances cell radioresistance.

## 5 Targeting Autophagy to Refine RT

Recently, various preclinical models have revealed that autophagy is activated in irradiated tumour cells, and inhibition of autophagy genes alone leads to a significant sensitising effect in radiation [91]. Therefore, the use of autophagy inhibitors is the most common strategy to target autophagy for RT. At present, several clinical trials have begun to combine autophagy inhibitors with cancer‐promoting death compounds to enhance the efficacy of RT. For example, hydroxychloroquine (HCQ) is a less toxic derivative of chloroquine (CQ), which inhibits autophagy by preventing lysosomal acidification and has been shown to be associated with enhanced cell radiosensitivity [104]. The focus of this strategy is to regulate autophagy in the late stages of cancer development or to prevent its progression to invasive and metastatic diseases. In contrast, several studies have pointed out that the combined application of autophagy promoters, such as rapamycin and its analogues, can also enhance the sensitivity of tumour cells to radiation.

It is worth noting that although autophagy modulators are the most tried adjuvants of targeting autophagy in cancer, their effectiveness is inevitably limited due to cancer heterogeneity [50]. For cancers with higher levels of autophagy, such as certain subtypes of breast cancer [72], RT could act synergistically with autophagy inhibitors to block the ability of autophagy as a survival pathway, thereby significantly enhancing therapeutic efficacy. However, when the cancer itself exhibits low levels of autophagy, for instance in triple‐negative breast cancer, some NSCLC [70], and pancreatic cancer [105], further inhibition of autophagy may not yield the desired effect. In such cases, inducing autophagy might turn out a favourable strategy, that is, to enhance the efficacy of RT by promoting autophagy‐dependent cell death. Overall, autophagy‐targeted RT should be individualised, taking into account tumour types, autophagy levels, patient condition, and treatment response to achieve an optimal therapeutic outcome.

In recent years, some RT therapies targeting other pathways to indirectly regulate autophagy have been emerging. The research status of targeted autophagy to improve the efficacy of RT is shown in Table 1.

**TABLE 1:** Strategies for targeting autophagy in RT.

Therapy	Mechanism	Cancer type	Ref.
Rapamycin, histone deacetylase inhibitors and suberoylanilide hydroxamic acid combination therapy	Increase the number of autophagosomes; increase the expression of autophagy markers and Atg5 protein; reduced p62 protein expression	NSCLC	[70]
Gemcitabine‐radiation targeted ATAD2 combined treatment	Increased levels of LC3l and autophagy markers; induce the expression of DNA damage marker gH2AX; down‐regulated the expression of DNA damage repair proteins pChk1 and pChk2	Pancreatic	[106]
Metformin	Inhibition of Gln metabolism and autophagy	Prostate	[93]
Up‐regulation of miR‐200c combined with carbon ion beam irradiation	Promote Bax expression to induce apoptosis, increase Beclin‐1 and p62 levels, and induce autophagy to inhibit tumour stem cells	Pancreatic	[105]
Anti‐aging flavonoids	Activation of autophagy through AMPK/MAPK pathway promotes apoptosis of human radiation‐resistant colon cancer cells	Colon	[107]
3‐methyladenine	Inhibition of autophagy by 3‐MA enhanced the expression of FOXO3 A transcription factor and PUMA	Osteosarcoma	[108]
Mitochondrial targeted antioxidant MitoQ	Pseudo‐mitochondrial membrane potential (PMMP) was constructed to inhibit mitochondrial respiration in normal cells and selectively induce autophagy in normal cells, which enhanced the radiation resistance of normal cells, but did not affect the tumour killing effect of radiation	Breast	[1]

## 6 Concluding Remarks and Future Prospects

From the above analysis, it can be concluded that there is still no consensus reached on the specific role of autophagy in the regulation of radiosensitivity, which calls for comprehensive discussion in combination with various factors such as tissue type and progression stage. However, regardless of the conditions, the results are highly correlated with the inducing factors of autophagy and the biological events activated by autophagy downstream. As mentioned above, previous studies have shown that autophagy can be widely involved in tumour glycolysis metabolism and can profoundly alter the metabolism of special tumour‐related amino acids such as glutamine, thereby exerting a regulatory effect on radiosensitivity. In recent years, lipid metabolism has been found to play an extremely critical role in tumour development and radiosensitivity regulation. However, the effect and molecular mechanism of autophagy mediated by lipid metabolism on radiosensitivity have not been well elucidated, which needs to be further explored in the future. Therefore, despite existing challenges, elucidating the regulatory role of autophagy in tumour radiosensitivity and its underlying mechanisms still represents a research avenue of both fundamental importance and considerable therapeutic potential.

## Author Contributions

All authors contributed to the study conception and design. H.L. conceived the review framework. H.L., Y.X. and C.D. contributed to the initial drafting of the manuscript and comprehensive editing. K.C. and X.X. assisted in data analysis, visualisation and manuscript refinement. X.H. participated in critical revision of the manuscript and provided intellectual feedback. J.C. and J.G. secured funding, oversaw the entire project, coordinated review development, and led revisions of the final manuscript. J.C. and X.H. jointly supervised the research and contributed to editorial oversight. J.G., as the lead corresponding author, managed correspondence, project administration, and ensured the integrity of the review process. All authors reviewed and approved the submitted version.

## Conflicts of Interest

The authors declare no conflicts of interest.

## Data Availability

Data sharing not applicable to this article as no datasets were generated or analysed during the current study.
